# Phase 1 trial of apatinib combined with intensity-modulated radiotherapy in unresectable hepatocellular carcinoma

**DOI:** 10.1186/s12885-022-09819-3

**Published:** 2022-07-15

**Authors:** Hongzhi Wang, Xianggao Zhu, Yuting Zhao, Dezuo Dong, Lijuan Li, Yong Cai, Yongheng Li, Weihu Wang

**Affiliations:** Key Laboratory of Carcinogenesis and Translational Research (Ministry of Education/Beijing), Department of Radiation Oncology, Peking University Cancer Hospital and Institute, No. 52 Fu-cheng Road, Haidian District, Beijing, 100142 People’s Republic of China

**Keywords:** Unresectable hepatocellular carcinoma, Apatinib, maximum tolerated dose, Intensity-modulated radiotherapy

## Abstract

**Background:**

To investigate the maximum tolerated dose (MTD) of apatinib delivered during and after intensity-modulated radiotherapy (IMRT) for unresectable hepatocellular carcinoma (HCC).

**Methods:**

Patients with unresectable HCC who were not eligible for radiofrequency ablation (RFA), transcatheter arterial chemoembolization (TACE), or residual/ recurrent after the prior local treatment were enrolled. Patients were scheduled to be treated with IMRT at 50–60 Gy/25–30 fractions. Oral apatinib tablets were administered concurrently with IMRT and continued thereafter. We used a 3 + 3 dose-escalation design, with three dose levels of apatinib (250, 500, and 750 mg). Grade 3 or more severe adverse events (AEs) were defined as dose-limiting toxicities (DLTs). The treatment response was calculated using the Modified Response Evaluation Criteria in Solid Tumours.

**Results:**

Nine patients with Barcelona Clinic Liver Cancer Stage C were included. One patient withdrew from the apatinib 250 mg group and another patient was added. No DLTs occurred in the apatinib 250 mg group. Five patients were included in the apatinib 500 mg group, and 2 cases of DLT (grade 3 leukopenia) were found among them. Dose escalation was terminated and the MTD was determined to be 250 mg. Common grade 1–2 AEs included fatigue, hypertension, dizziness, bone marrow suppression, and hyperbilirubinemia. The median follow-up time for all patients was 16.0 months. Three patients achieved complete response and another three achieved partial response. The objective response rate was 6/9 (66.7%), and the disease control rate was 9/9 (100%). Three patients relapsed out of the radiation field. The median progression-free survival was 17.0 months, and the median overall survival was 16.7 months.

**Conclusions:**

When combined with IMRT, apatinib 250 mg daily was recommended for a phase 2 study of unresectable HCC. The antitumor activity of the combination treatment was encouraging. The safety and efficacy of apatinib combined with IMRT for unresectable HCC should be further investigated in future studies.

**Trial registration:**

Registration No. ChiCTR1800018309. Registered 11 September 2018. Retrospectively registered, https://www.chictr.org.cn/showproj.aspx?proj=30461.

**Supplementary Information:**

The online version contains supplementary material available at 10.1186/s12885-022-09819-3.

## Background

Liver cancer is the fourth most common cancer and the second leading cause of cancer-related mortality in China [1, 2]. Hepatocellular carcinoma (HCC) is the most common pathological pattern of primary liver cancer, accounting for 75–85% of cases [3]. Most patients with liver cancer are asymptomatic and typically unresectable when first diagnosed. Advances in radiotherapy techniques, such as three-dimensional conformal radiotherapy, intensity-modulated radiation therapy (IMRT), and stereotactic body radiotherapy, have allowed for enhanced delivery of higher doses to the tumour while sparing normal liver tissue [4–7]. Radiotherapy has become an important choice for the locoregional treatment of HCC. However, intrahepatic metastasis outside the radiation field is usually identified as the first failure [8]. Thus, a treatment strategy that combines radiotherapy with systemic therapy may be recommended.

Apatinib is a small-molecule receptor tyrosine kinase inhibitor (TKI) that displays potent inhibitory activity against multiple tyrosine kinases such as vascular endothelial growth factor receptor-2 [9]. Apatinib has been demonstrated to exert potential antitumor activity in multiple solid tumours, such as gastric cancer, ovarian cancer, HCC, colorectal cancer, lung cancer, and osteosarcoma [10–14]. In a placebo-controlled, double-blind, phase 3 clinical study, apatinib as second-line therapy in Chinese patients with advanced HCC showed an increased objective response rate (ORR; 11% vs. 2%), median progression-free survival (mPFS; 4.5 vs. 1.9 months), and median overall survival (mOS; 8.7 vs. 6.8 months) compared to the placebo group [15]. In a randomised phase 2 clinical study, apatinib in combination with transcatheter arterial chemoembolization (TACE) showed an excellent PFS benefit compared to TACE alone (mPFS: 12.5 vs. 6.0 months) in the treatment of HCC [16]. Thus, apatinib is an effective systemic therapy for HCC treatment when used alone or in combination with TACE.

Here, we speculated that apatinib combined with radiotherapy may be an effective therapeutic regimen. However, the safety of this HCC treatment has not yet been investigated. Therefore, we undertook this dose-escalating study to determine the safe dose of apatinib when combined with IMRT in the treatment of patients with unresectable HCC.

## Methods

### Patients

Eligible patients were aged between 18 and 75 years with an Estern Cooperative Oncology Group performance score of 0–1. HCC was diagnosed based on a biopsy specimen of the tumour, or imaging criteria (CT/MRI LI-RADS v2017) [17]. Patients with HCC were unresectable or relapsed after surgery and not suitable for re-operation. Patients were not suitable for radiofrequency ablation (RFA) or residual/recurrent after RFA. Patients were not suitable for TACE or had no substantial necrosis after TACE treatment. Patients were required to have > 700 mL of uninvolved liver with Child–Pugh class A. The white blood cell count was ≥ 3.0 × 10^9^/L, neutrophils count ≥ 1.5 × 10^9^/L, platelet count ≥ 75 × 10^9^/L, bilirubin < 1.5 × upper limit of the normal value (ULN), and alanine transaminase and aspartate transaminase < 2.5 × ULN. Patients infected with hepatitis B virus (HBV) must have had HBV DNA levels < 500 IU/mL. The exclusion criteria were as follows: apatinib allergy; previous systemic therapy history; extrahepatic metastasis; pregnant or lactating women, or women of child-bearing age who did not use adequate contraception; untreated or incompletely treated medical conditions, such as uncontrolled hypertension and diabetes; human immunodeficiency virus positive; bleeding or clotting disorder; stroke or myocardial infarction within 6 months; and gastroduodenal ulcer or upper gastrointestinal bleeding within 3 months.

In this phase 1 study, a traditional 3 + 3 dose escalation design was used. Apatinib and IMRT were administrated on day 1, and apatinib treatment continued after IMRT until the tumour progressed or intolerant toxicity was observed. IMRT in combination with three different dose levels of apatinib (250 mg daily, 500 mg daily, and 750 mg daily) were planned for each group. The apatinib dose was escalated if none of the three patients experienced dose-limiting toxicity (DLT) within 16 weeks after IMRT initiation. If one of the three patients developed DLT, another three patients were recruited to the same dose group. When two or more patients out of the six experienced DLTs in a dose level group, the prior dose level was considered as the maximum tolerated dose (MTD). This study was approved by the Peking University Cancer Hospital Ethics Committee (Beijing, China), and all the patients provided written informed consent. The study was retrospectively registered at www.chictr.org.cn (Registration No. ChiCTR1800018309).

### Radiotherapy

Simulating computed tomography (CT) and magnetic resonance imaging (MRI) scans were performed with patients in the supine position, along with thermoplastic mask immobilisation. Image registration was performed between simulating CT and MRI to optimise the target and normal structure delineation using the Eclipse treatment planning system (Varian Medical Systems, Palo Alto, CA, USA). IMRT planning with 6–10 MV X-rays was performed. The prescription dose was 50–60 Gy in 25–30 fractions. The prescribed dose of radiotherapy was based on the upper limit of dose distribution of normal liver tissue and surrounding organs. The dose constraints of organs at risk (OARs) were as follows: mean dose (D_mean_) of normal liver volume < 24 Gy, D_mean_ of kidney < 15 Gy, maximum dose (D_max_) of stomach < 54 Gy, D_max_ of small intestine < 54 Gy, and D_max_ of spinal cord < 45 Gy.

### Safety and Response Evaluation

The severity of adverse events (AEs) was assessed using the National Cancer Institute Common Terminology Criteria for Adverse Events, version 4.0. Grade 3 or more severe AEs in the first 16 weeks after IMRT initiation were defined as DLT [18]. Grade 3 hypertension was not defined as DLT if it could be controlled to grade 0–2 by antihypertensive drugs [19]. The cumulative toxicities from extended treatment cycles were also monitored. The treatment response was evaluated using the Modified Response Evaluation Criteria in Solid Tumours (mRECIST) [20]. CT scans of the chest, abdomen, and pelvis, as well as MRI of the liver were performed at baseline, 4 weeks after IMRT, and then every 8–12 weeks.

### Statistics analysis

Continuous variables were presented as median (range), while categorical variables were presented in terms of number and percentage. The Kaplan–Meier method was used to calculate the time to progression and survival. OS was defined as the time from the start of treatment to death from any cause or to the last follow-up. PFS was defined as the time from the start of treatment to disease progression or death. Statistical analyses were performed using IBM SPSS Statistics, version 22.0 software (Armonk, NY, USA).

## Results

### Patient characteristics

Nine patients with Barcelona Clinic Liver Cancer Stage C stage were enrolled between January 2018 and November 2019. Eight patients had portal vein tumour thrombosis, and one patient displayed invasion of the inferior vena cava but without thrombosis. None of the patients had extrahepatic diseases. All nine patients were men, with a median age of 50 years (range: 46–72 years). The baseline characteristics of the patients are presented in Table 1.

**Table 1:** Baseline characteristics of patients

**Clinical Characteristics**		**Number (%)**
**Age (y)**	Median (range)	50 (46–72)
**Sex**	Male	9 (100.0)
	Female	0 (0)
**Hepatitis virus**	HBV infection	9 (100.0)
	HCV infection	0 (0)
**Tumour number**	Median (range)	1 (1–2)
**Tumour size (cm)**	Median (range)	5.0 (1.3–13.2)
**Tumour thrombosis**	PVTT	8 (88.9)
	IVCTT	0 (0)
**Previous therapy**	Surgery	1 (11.1)
	TACE	7 (77.8)
	RFA	2 (22.2)
	Systemic therapy	0 (0)

*Abbreviations*: *HBV* Hepatitis B virus, *HCV* Hepatitis C virus, *PVTT* Portal vein tumour thrombosis, *IVCTT* Inferior vena cava tumour thrombosis, *TACE* Transarterial chemoembolization, *RFA* Radiofrequency ablation

### Treatment and dose escalation

In the apatinib 250 mg group, one patient (case 2) withdrew five weeks after the start of treatment due to planning to receive TACE therapy. The duration of apatinib treatment was 1.0 month, and no DLT was observed. Therefore, another patient was added (case 4) to the apatinib 250 mg group. All three patients (cases 1, 3, and 4) completed the planned treatment, and no DLT occurred during the observation period.

The other three eligible patients (cases 5–7) were included in the apatinib 500 mg group, among which one patient (case 7) developed DLT (grade 3 leukopenia) 3 weeks after receiving treatment. Two additional patients (cases 8 and 9) were enrolled in this group. After five weeks of treatment, DLT (grade 3 leukopenia) occurred in case 9, indicating that DLT occurred in two out of the five patients in this group. Three weeks after stopping apatinib treatment in these two patients, their white blood cell counts gradually recovered to grade 0–1. Dose escalation was terminated and the MTD was determined to be 250 mg.

### Safety

All nine patients were included in the safety analysis, as shown in Table 2. Within the 16 weeks of treatment, the most common AEs were hyperbilirubinemia (4/4) and hypertension (4/4) in the apatinib 250 mg group, including one case of grade 3 hypertension, which could be controlled to grade 1 with antihypertensive drugs. In the apatinib 500 mg group, leukopenia, neutropenia, and hypertension were observed in all five cases. Other common AEs included thrombocytopenia (4/5), hyperbilirubinemia (4/5), dizziness (4/5), fatigue (4/5), nausea (4/5), proteinuria (3/5), headache (3/5), and hand-foot syndrome (3/5).

**Table 2:** Treatment-related toxicities for each dose cohort during the first 16 weeks of treatment

**Adverse events**	**IMRT + apatinib 250 mg**			**IMRT + apatinib 500 mg**		
	Grade 1	Grade 2	Grade 3	Grade 1	Grade 2	Grade 3
**Leukopenia**	1	2	0	0	3	2
**Neutropenia**	2	0	0	2	3	0
**Anaemia**	1	0	0	1	0	0
**Thrombocytopenia**	0	1	0	0	4	0
**ALT increased**	2	0	0	2	0	0
**AST increased**	1	1	0	2	0	0
**Hyperbilirubinemia**	2	2	0	3	1	0
**Hypoalbuminemia**	3	0	0	0	1	0
**Proteinuria**	0	0	0	2	1	0
**Headache**	0	0	0	2	1	0
**Dizziness**	1	2	0	3	1	0
**Fatigue**	3	0	-	3	1	-
**Nausea**	2	0	0	3	1	0
**Diarrhoea**	0	0	0	1	0	0
**Hand-foot syndrome**	0	0	0	2	1	0
**Hypertension**^**a**^	2	1	1	1	1	3

*Abbreviations*: *IMRT* Intensity-modulated radiation therapy, *ALT* Alanine aminotransferase, *AST* Aspartate aminotransferase

^a^ One case of grade 3 hypertension in the apatinib 250 mg group and 3 cases of grade 3 hypertension in apatinib 500 mg group were found, all of which could be controlled to grade 0–1 and were not defined as dose-limiting toxicities in this combination treatment regimen

The median apatinib administration time was 7.4 (1.0–10.9) months in the 250 mg group and 6.6 (1.1–14.2) months in the 500 mg group. In the subsequent apatinib treatment of the 500 mg group, two patients presented with severe AEs. One case of liver decompensation occurred within 6.6 months. The patient presented with hypoalbuminemia and ascites, then died of hepatic encephalopathy. Another patient developed upper gastrointestinal haemorrhage within 5.3 months, but the bleeding was controlled by symptomatic and supportive treatment. No severe AEs were found in the subsequent apatinib treatment of the 250 mg group.

### Treatment response and survival

**Fig. 1:**
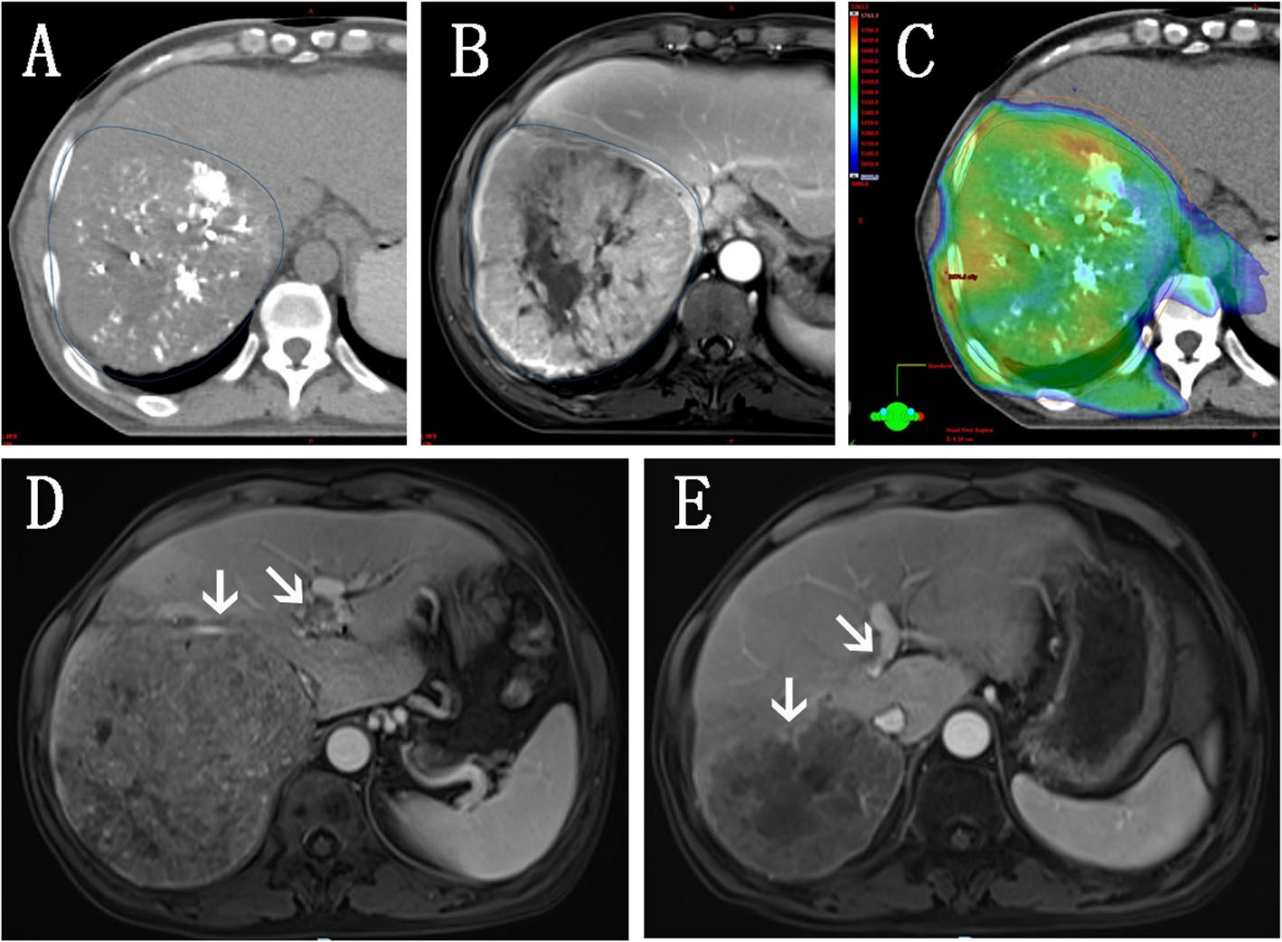
Implementation of radiotherapy and treatment response for patient case 4; **A** GTV delineation in simulation CT image; **B** GTV delineation in simulation MR image; **C** Dose distribution in the radiation plan, protecting normal liver tissue and digestive tract as much as possible; **D** Primary tumour in right lobe of liver and portal vein tumour thrombosis in the pre-treatment MR image; **E** Four weeks after the combined treatment of radiation and apatinib, the primary tumour and tumour thrombus reduced obviously, and partial response was achieved according to mRECIST. (white arrow, primary tumour and portal vein tumour thrombus)

In this study, three patients achieved complete response, while three more achieved partial response. The remaining three patients maintained stable diseases status. The ORR was 6/9 (66.7%), and the disease control rate was 9/9 (100%). Figure 1 shows one case of implementation of radiotherapy and treatment response after combined treatment with apatinib. The median follow-up time for all patients was 16.0 (range: 6.0-28.0) months. Cases 1, 5, and 8 were relapsed out of the radiation field in 10.2 months, 23.2 months, and 5.5 months, respectively. The median PFS was 17.0 months, and the median OS was 16.7 months. Details of tumours and treatments for each patient are shown in supplementary table 1. Dose distributions of OARs in radiotherapy are shown in supplementary table 2.

## Discussion

In this dose-escalating study of patients with unresectable HCC, two DLT cases (grade 3 leukopenia) were observed in the apatinib 500 mg group. Therefore, in combination with IMRT, apatinib 250 mg daily was considered as the recommended dosage in the phase 2 study.

Hypertension was a commonly observed AE in previous studies of apatinib treatment, and the incidence of hypertension was 40% in the treatment of metastatic gastric cancer [14] and 73% in HCC [21]. Considering that hypertension typically occurs early after apatinib treatment and can be well controlled by antihypertensive agents, well-controlled hypertension was not defined as a DLT in this study [19]. Four cases in our study were found to have grade 3 hypertension in the first week of treatment with apatinib, and all cases of hypertension were controlled to grade 0 or 1 through single or combined antihypertensive drugs.

In the AHELP study, apatinib alone as second-line therapy in advanced HCC, a dose of 750 mg once daily was administrated. Outcome showed that 77% of the patients exhibited grade 3–4 treatment-related adverse events (TRAEs). Neutropenia (11%) was common, which was more frequent than that in the RESORCE (regorafenib treatment for HCC who progressed on sorafenib) trial [15]. The median exposure duration of apatinib was 3.6 months. The treatment interruption and dose modification due to TRAEs were found in 60% and 45% of patients, respectively. A standard dose of 750 mg daily was difficult to maintain long-term.

It has been reported that in the treatment of locally advanced HCC with radiotherapy, the main treatment-related grade 3 toxicities were leukopenia (17%) and thrombocytopenia (13%) [8]. When radiotherapy is combined with apatinib in treatment of HCC, there are reasons to expect that increments of hematological toxicity will be found. Actually, in our dose-escalating study, the DLT was leukopenia and in the long-term treatment and follow-ups, leukopenia and thrombocytopenia were common. Similarly, a superposition of toxicity was found in treatment of TACE combined with apatinib; the dose of apatinib was adjusted to 250–500 mg daily [16]. With the advent of immunotherapy, its combination with TKIs has shown excellent efficacy in patients with advanced HCC. In the study of camrelizumab (immune checkpoint inhibitors) combined with apatinib in treatment of advanced HCC (RESCUE trial) [21], the dose of apatinib was set as 250 mg once daily; the common treatment-related adverse events of ≥ grade 3 were hypertension, neutropenia, thrombocytopenia, increased AST, and hyperbilirubinemia. In combination therapy with different regimens, superposition of toxicity was frequently found.

The OARs in radiotherapy for HCC included the spinal cord, normal liver tissue, gastrointestinal tract, etc. We should first ensure the safety of the OARs and then increase the radiation dose as much as possible. This study strictly limited the doses distributed to OARs and the dose of the target volume was defined to 50–60 Gy, 25–30 fractions. The heterogeneity of radiotherapy was well controlled in terms of the radiotherapy technique, target delineation principles, prescribed doses, and OARs dose limitations. The MTD of apatinib was explored on the premise of the safe implementation of radiotherapy. Therefore, when combined with IMRT, the dose of apatinib was recommended to be 250 mg daily in the treatment of locally advanced HCC.

In this study, AEs of grade 1–2 were common, such as fatigue, hypertension, dizziness, bone marrow suppression, and hyperbilirubinemia. Even though grade 1–2 toxicities are usually reversible, treatment-associated toxicities should be taken seriously because of the typical patient histories of hepatitis or cirrhosis. In the subsequent course of apatinib treatment, one case of liver decompensation occurred within 6.6 months, and the patient died of hepatic encephalopathy. Another patient developed upper gastrointestinal haemorrhage within 5.3 months, but the bleeding was controlled by symptomatic treatment. Given the comorbidities of liver cirrhosis, the occurrence of severe AEs and decompensation-related deaths should be considered in HCC treatment. We thought it important in locally advanced HCC to ensure the safety of radiation therapy and to ensure the tolerance in long-term targeted therapy.

The outcome of systemic treatment alone for HCC was unsatisfactory. In the standard first-line treatment, lenvatinib and sorafenib showed similar survival, and the median survival time was 13.6 months and 12.3 months, respectively [22]. According to the mRECIST evaluation criteria, the ORR and mPFS were 40.6% and 7.3 months, respectively, in the lenvatinib group, and 12.4% and 3.6 months respectively in the sorafenib group. Systemic therapy combined with effective locoregional therapy is a promising approach in locally advanced HCC. In a previous study, apatinib was shown to be effective in combination with TACE, with the best ORR and mPFS of 60% and 12.5 months, respectively [16]. Similarly, in this study, apatinib combined with IMRT for the treatment of locally advanced HCC showed an encouraging outcome, where the best ORR was 67%, the mPFS was 17.0 months, and the mOS was 16.7 months. In a previous study by our team, 63 patients with HCC and macrovascular invasion, underwent IMRT plus TACE combined with or without sorafenib from 2015–2018 [8]. In the failure pattern analysis, intrahepatic metastasis out of the radiation field was the most common failure in the locoregional treatment group, with an incidence of 57.1%. However, in the locoregional treatment plus sorafenib group, intrahepatic metastasis decreased to 28.6%. Thus, locoregional treatment combined with systemic treatment may be an effective treatment option for locally advanced HCC.

## Conclusions

In summary, we reported that apatinib 250 mg daily may be a safe dosage when combined with IMRT for the treatment of unresectable HCC. The antitumor activity of the combination regime was encouraging. However, due to the small sample size, the efficacy reported in this phase 1 study should be interpreted with caution. The safety and efficacy of apatinib combined with IMRT for unresectable HCC should be investigated in future studies.

## Supplementary Information

**Additional file 1:** **Supplementary Table 1** Details of tumours and treatments for each patient. **Supplementary Table 2** Dose distribution of digestive tract and normal liver tissue in the treatment of radiotherapy.

**Additional file 2.** CONSORT 2010 checklist of information to include when reporting a randomised trial*.

## Data Availability

The statistical datasets and codes used and/or analyzed in the current study are available from the corresponding author (wangweihu88@163.com) on reasonable request.

## Abbreviations

AEs: Adverse events
CT: Computed tomography
DLTs: Dose-limiting toxicities
Dmax: Maximum dose
Dmean: Mean dose
HBV: Hepatitis B virus
HCC: Hepatocellular carcinoma
IMRT: Intensity-modulated radiotherapy
LI-RADS: Liver Imaging Reporting and Data System
mOS: Median overall survival
mPFS: Median progression-free survival
mRECIST: Modified Response Evaluation Criteria in Solid Tumours
MRI: Magnetic resonance imaging
MTD: Maximum tolerated dose
ORR: Objective response rate
RFA: Radiofrequency ablation
TACE: Transcatheter arterial chemoembolization
