# Supplementary material for: Phase 1 trial of apatinib combined with intensity-modulated radiotherapy in unresectable hepatocellular carcinoma
Source: BMC Cancer. 2022 Jul 15;22:771. doi: 10.1186/s12885-022-09819-3 (PMC9287866; doi:10.1186/s12885-022-09819-3)
Supplement: Supplementary file 1 — Additional file 1: Supplementary Table 1 Details of tumours and treatments for each patient. Supplementary Table 2 Dose distribution of digestive tract and normal liver tissue in the treatment of radiotherapy. [file 12885_2022_9819_MOESM1_ESM.docx]

Supplementary Table 1. Details of tumours and treatments for each patient.

| Patients | Number of Tumours | Location of primary Tumours | Size of Tumours (cm) | Tumour Thrombosis | Dose of Radiotherapy | Duration of Apatinib (mo) | Response |
| --- | --- | --- | --- | --- | --- | --- | --- |
| case 1 | 2 | s6 | 8.1 | portal vein | 56gy/28f | 10.2 | CR |
|  |  | s7 | 6.2 |  |  |  |  |
| case 2 | 1 | s4 | 6.4 | portal vein | 50gy/25f | 1.0 | SD |
| case 3 | 1 | s6, s7 | 3.8 | portal vein | 56gy/28f | 12.0 | PR |
| case 4 | 1 | s6, s7, s8 | 13.2 | portal vein | 50gy/25f | 4.5 | PR |
| case 5 | 1 | s6, s7 | 8.3 | portal vein | 50gy/25f | 9.0 | CR |
| case 6 | 1 | s7, s8 | 5.0 | none | 60gy/30f | 16.0 | CR |
| case 7 | 2 | s7 | 1.3 | portal vein | 54gy/27f | 6.6 | SD |
|  |  | s7 | 3.3 |  |  |  |  |
| case 8 | 2 | s7 | 3.7 | portal vein | 50gy/25f | 5.3 | PR |
|  |  | s4 | 1.3 |  |  |  |  |
| case 9 | 2 | s6, s7, s8 | 5.0 | portal vein | 50gy/25f | 1.2 | SD |
|  |  | s1 | 3.9 |  |  |  |  |

CR complete response, PR partial response, SD stable disease

Supplementary Table 2. Dose distribution of digestive tract and normal liver tissue in the treatment of radiotherapy.

| Patients | Digestive Tract (Dmax) | | | | Normal Liver Tissue (Volume, Dmean) | |
| --- | --- | --- | --- | --- | --- | --- |
|  | Esophagus (Gy) | Stomach (Gy) | Duodenum (Gy) | Other Small Intestine (Gy) | Volume (ml) | Dmean (Gy) |
| case 1 | 16.7 | 45.0 | 54.0 | 22.8 | 1425 | 23.3 |
| case 2 | 51.2 | 54.0 | 54.0 | 54.0 | 1216 | 21.9 |
| case 3 | 24.0 | 51.8 | 32.0 | 54.0 | 786 | 22.5 |
| case 4 | 52.7 | 54.0 | 53.2 | 53.8 | 989 | 21.7 |
| case 5 | 53.4 | 41.2 | 54.0 | 55.0 | 724 | 19.7 |
| case 6 | 53.8 | 45.2 | 53.6 | 38.6 | 1169 | 22.3 |
| case 7 | 53.6 | 54.5 | 54.5 | 41.2 | 1216 | 21.1 |
| case 8 | 39.6 | 33.6 | 53.0 | 20.2 | 1213 | 21.0 |
| case 9 | 54.8 | 53.7 | 53.9 | 46.9 | 1201 | 21.1 |

D_mean_ mean dose, D_max_ maximum dose.
